# Patient and public involvement prior to trial initiation: lessons learnt for rapid partnership in the COVID-19 era

**DOI:** 10.1186/s40900-021-00250-9

**Published:** 2021-03-08

**Authors:** Zahra Jamal, Alexander Perkins, Christopher Allen, Research Advisory Group, Richard Evans, Joanna Sturgess, Claire Snowdon, Tim Clayton, Diana Elbourne

**Affiliations:** 1 London School of Hygiene & Tropical Medicine, Keppel Street, London, WC1E 7HT UK; 2 St Thomas’ Hospital, Westminster Bridge Rd, Bishop’s, London, SE1 7EH UK

**Keywords:** Patient and public involvement, Clinical trials, COVID-19

## Abstract

**Plain English summary:**

Patient and Public Involvement (PPI) describes the active involvement of patients and the public in the research process. Through PPI, patients and members of the public are increasingly involved in the design and conduct of clinical trials. PPI has been shown to improve the quality and relevance of research.

During the COVID-19 pandemic, clinical trials have been playing a vital role in helping us find ways to prevent and treat the infection and improve our understanding of the virus. It is important that patients and the public are actively involved in deciding how COVID-19 research is carried out. Unfortunately, Research Ethics Committees in the UK have seen far less PPI for COVID-19 research studies compared with research before the pandemic. A key reason for this is that research is being designed much faster than normal and researchers may feel they do not have time to properly involve patients and the public.

In this paper, we share our experiences of PPI for a COVID-19 clinical trial. We show that it is possible to rapidly involve patients and the public in COVID-19 clinical trials. We also explain how the design of the clinical trial was changed in response to feedback from public contributors. Lastly, we discuss the wider learning from this process which might be useful for researchers planning PPI activities for COVID-19 clinical trials in the future.

**Abstract:**

**Background:**

Clinical trials are playing a critical role in the global public health response to the COVID-19 pandemic. Despite the increasing recognition of the value of PPI in clinical trials, just 22% of the COVID-19 research proposals reviewed by Research Ethics Committees in the UK at the start of the pandemic reported PPI. There is a perception that PPI might result in delays in delivering research and therefore delays in obtaining important results. In this paper, we report our experience of rapid PPI for a COVID-19 clinical trial.

**Methods:**

RAPID-19 is a COVID-19 clinical trial which was planned to be submitted for fast-track ethics review in the United Kingdom. During the development of the trial protocol, the PPI Panel at the London School of Hygiene & Tropical Medicine Clinical Trials Unit was involved in the design of the study. The meeting with the PPI Panel lasted just over 1 h and was conducted by teleconference.

**Results:**

Although we only had a short period of time to explore the study with the PPI Panel, we were able to gain valuable insight into how the trial would be perceived by potential trial participants. Substantive changes were made to the trial to improve the acceptability of the research without compromising the study timelines. Having access to public contributors with relevant lived experience is an important resource for a Clinical Trials Unit and is critical for rapid PPI. The move to remote working due to lockdown required virtual discussions which helped to overcome some of the barriers to organising face-to-face meetings at short notice.

**Conclusions:**

PPI for clinical trials can be conducted in a time-efficient manner within the pressured environment of a pandemic. Involving PPI contributors at an early stage in protocol development maximised the opportunity to shape and influence the trial as well as limited potential delays which could occur if changes to the protocol had to be made at a later stage.

**Supplementary Information:**

The online version contains supplementary material available at 10.1186/s40900-021-00250-9.

## Introduction

Patient and Public Involvement (PPI) in research describes research which is “being carried out ‘with’ or ‘by’ members of the public” not just “‘to’, ‘about’ or ‘for’ them” [1]. There are compelling moral and practical arguments for promoting active partnerships between researchers and members of the public. The moral argument is that patients and the public have a right to be involved in research that affects them. From a practical perspective, PPI is understood to afford insights to researchers that would not otherwise be available, in order to improve the quality and relevance of studies.

PPI in clinical trials encompasses a range of activities and types of involvement which can occur across all stages of a study from conception to dissemination. There is increasing recognition of the value of PPI in clinical trials. A recent systematic review found that PPI is likely to improve participant recruitment levels for clinical trials [2].

Designing and conducting clinical trials in the context of the COVID-19 pandemic presents many challenges which include ensuring the active involvement of patients and the public in the research process. It is important that these challenges are addressed, as research is playing a key role in the global public health response to the pandemic. Well-conducted, high quality clinical trials are critical to both our understanding of the virus and in the development of diagnostic, prophylactic and therapeutic products.

The crisis has sparked a wave of research activity. At the time of submission of this paper, there have been 2489 COVID-19 studies registered on ClinicalTrials.gov since the start of the pandemic [3]. In the UK, this has been made possible through the development of new procedures to provide swift reviews by Research Ethics Committees for COVID-19 research. As a result, studies can be reviewed within 24 h of submission [4].

It is important that with expedited trial design and approvals, PPI in clinical trials is not neglected. Only 22% of the COVID-19 research proposals submitting to Research Ethics Committees (REC) in the UK in March 2020 included PPI compared with 80% of research reviewed prior to the start of the pandemic [5].

The experience of conducting research during the 2014–15 West African Ebola epidemic showed that failure to engage appropriately with communities led to distrust and community backlash which, in turn, hindered early research efforts [6]. On the other hand, an understanding of the concerns of communities and an emphasis on building relationships with them based on trust, mutual respect and active involvement eventually helped to deliver successful Ebola trials [6].

The World Health Organization’s Good Participatory Practice Guidelines for Trials of Emerging (and Re-emerging) Pathogens emphasises the importance of investing time to effectively engage stakeholders in the research process [7]. However, the COVID-19 pandemic represents a time-critical situation. By July 2020, the UK had experienced one of the highest death tolls globally from COVID-19 [8]. The urgency of the situation demands that research seeking to answer key scientific questions in understanding and tackling the pandemic, including preparing for future outbreaks, be conducted swiftly and efficiently.

In this paper, we present an example of PPI prior to a COVID-19 clinical trial in the UK.

### The clinical trial

Patients with cardiovascular disease, high blood pressure and diabetes are at particularly high risk of poor outcomes from COVID-19 [9]. One possible explanation for this increased risk lies in the medications used in the management of these health conditions. ACE-inhibitors(ACEi) and angiotensin receptor blockers (ARBs) are medications commonly used to treat high blood pressure, diabetes and heart failure. These medications increase the expression of the receptor used by the COVID-19 virus to enter cells in the body. However, the mechanism of this interaction in influencing the severity of illness with COVID-19 is not understood.

RAPID-19 is a pilot open-label randomised trial to establish whether ACEi and ARBs affect the prognosis and severity of illness in ambulatory patients with COVID-19. Patients on ACEi/ARBs who present at hospital with suspected or confirmed COVID-19, and are considered well enough to return home to recover from their COVID-19 symptoms, are eligible to take part in the study. Participants with a confirmed diagnosis of COVID-19 are randomised to either continue taking their medications (control group) or to temporarily withdraw ACEi or ARB for 14 days. Temporary withdrawal of these medications is not associated with clinically significant increases in blood pressure and participants will be monitored for safety concerns. Participants are asked to complete a daily diary for 14 days to monitor their symptoms. They are also followed up by telephone at 7 days, 14 days, and 30 days post-randomisation. Researchers are available via telephone and in person to address concerns and record adverse events.

RAPID-19 is a collaboration between King’s Health Partners and the London School of Hygiene & Tropical Medicine (LSHTM) Clinical Trials Unit (CTU). The trial is sponsored by King’s College London. Although the trial was submitted for ethical approval and the application was granted a provisional opinion, the Trial Management Group decided not to proceed with opening the trial. Cases of COVID-19 had started to decline in the UK during the first wave of infection which would have hindered recruitment into the trial. In addition, other international trials examining the same research question had also been set-up and were already recruiting participants.

In accordance with the INVOLVE statement on public involvement in research and research ethics committee review [10], ethics approval was not sought for our public involvement work on the clinical trial.

## Methods

The RAPID-19 Trial Management Group (TMG) began to develop a protocol for the trial which they planned to submit for fast-track ethics review. The LSHTM CTU has an established cardiovascular disease (CVD) PPI panel (the Panel) comprising 10 members who have either lived experience of CVD, or have direct experience as a carer or family member to someone with CVD. The composition of the Panel is diverse in terms of gender and ethnicity and broadly reflective of the target population. The purpose of the Panel is to work with the CTU in the design of cardiovascular clinical trials to optimise the acceptability, impact and relevance of studies.

We had only two working days to explore the RAPID-19 Trial with the Panel and elicit feedback. It was agreed to hold a meeting by teleconference to meet the needs of all attendees. Prior to the meeting, the members were emailed a written summary of the proposed trial design. The meeting was attended by seven out of the ten members of the Panel and lasted just over 1 h. A topic guide was used to facilitate the discussions. The meeting was not audio-recorded. In a separate meeting, we also sought feedback on the trial from a patient representative on the National Cardiac Audit Programme. The feedback from the meeting was written up by sorting and arranging the comments from the Panel under thematic headings. Below we describe the insights offered by PPI consultation and the actions taken in response.

The Guidance for Reporting Involvement of Patients and the Public (GRIPP2) short form checklist was used to guide the reporting of PPI in the paper [11].

## Results

### Risk assessment

A key issue raised by the Panel related to the intervention itself. The intervention violates a common maxim of medical advice, that one should not abruptly stop taking prescribed medications without consulting a doctor. The contributors described their personal experiences of changing or stopping medications. They explained that this decision requires a discussion between their primary care doctor and hospital consultant to examine the impact and possible side effects of the regime change. A decision to stop a medication may require the dose to be reduced gradually over time.

In the trial scenario, the research staff may not have access to the complete medical history of patients they approach, and it may not always be practical to discuss their case with relevant specialists. The Panel explained that, in order to consider participating in the trial, they would need to feel confident that the attending clinician who offers recruitment into RAPID-19 has assessed their clinical history and that, taking into account all relevant information, the clinician is able to make an informed assessment as to their suitability for the trial.

### Clinical monitoring

PPI contributors remarked how the pandemic is creating an exceptional backdrop for the conduct of research. People are isolated from friends and family, some are fearful of attending hospitals and terrified as to how the virus may affect them. Participants will be feeling risk-averse and consequently need to feel assured that steps have been taken to mitigate any risks to which they could be exposed.

One concern raised by the Panel was that the level of clinical monitoring provided by the trial does not satisfactorily address the risk felt by participants told to stop taking their medication. For example, how would participants know if their blood pressure becomes dangerously high. There were mixed feelings about the usefulness of home blood pressure monitors being provided to participants. Some felt that recording daily blood pressure would be an additional burden and, even if training was provided on using the monitors, participants may still feel worried they are not using the device correctly.

### Adherence to treatment arm

Members of the Panel commented that some participants may find it difficult adjusting to the change in medication dictated by the intervention. One concern was that participants who receive their medications in pre-prepared dosette boxes from a pharmacy for multidrug regimens would need to identify and remove the correct tablet from their blister pack. It was thought that some patients accustomed to a set routine may forget that they are under a new, altered drug regime and forget to remove the tablet.

The Panel also discussed whether automated mobile phone messaging could be used to help participants adhere to their allocated treatment. It was felt that, in order to be effective, text messages would need to be sent to coincide with the times participants normally take their tablets and would therefore require customised messaging. The question was raised as to what to do for participants who do not have mobile phones.

### Response by TMG to evidence from the PPI panel

The TMG considered the evidence from the PPI panel and used the insights gained to adapt the trial protocol as follows:

#### Risk assessment

The TMG added a further exclusion criterion to the eligibility criteria. It supports the case for the attending clinician to assess, in conjunction with the patient and on the basis of the patient’s clinical history, whether their participation in the trial would put them at risk. Patients who are considered at significant clinical risk from participation in the trial would not be eligible to take part.

#### Clinical monitoring

The TMG developed a daily patient diary for participants to use to monitor their symptoms (see Additional file 1). The diary was provided to participants in paper format. The records have been designed to alert the participants if they experience any potentially harmful symptoms that develop as a consequence of stopping their medication. It is intended that these alerts would prompt participants to contact the clinical team if they experienced any worrying symptoms.

#### Adherence to treatment arm

The diary was also specifically designed to help participants remember a change in their medication by getting them to complete their diary at the same time each day. The diary asks participants if they have adhered to their treatment allocation. Furthermore, the TMG decided to exclude from the trial those patients who receive medication in a blister pack/dosette box.

Despite the short time-frame available, we were able to gain valuable insight into how the trial would be perceived by potential trial participants. The ability to feed the views of the PPI Panel directly into the protocol, with little delay to the REC application process, indicates that a responsive process is possible and appropriate.

## Discussion

Time constraint has been cited as a barrier to PPI in research during the pandemic [12]. The experience described in this paper demonstrates that valuable PPI for clinical trials can be conducted in a time-efficient manner within the pressured environment of a pandemic. The adaptations to the trial protocol, which were made as a consequence of the PPI work, were vital for improving the acceptability of the study to potential participants and which may in turn help to support recruitment and retention into the trial.

Key to this process was involving PPI contributors at a very early stage which maximised the opportunity to shape and influence the trial. This also limited potential delays which could occur if changes to the protocol had to be made at a later stage. Furthermore, conducting thorough PPI during the development of the protocol demonstrates to Research Ethics Committees that the safety and wellbeing of patients is being taken into consideration. This may help to reduce the number of revisions required by RECs improving the efficiency of the ethical review process (Health Research Authority: HRA COVID-19 Public Involvement workshop summary, unpublished).

The time required to identify appropriate public contributors to be involved in the research process at short notice can also be a barrier to PPI. In recognition of this issue, the Health Research Authority has launched a public involvement service for COVID-19 research studies applying for fast-track ethics review [13]. The service provides guidance to researchers as to who they should involve and what type of involvement they should seek, and then matches research teams with coordinators of relevant public involvement groups across the UK.

We were fortunate to already have access to an active PPI panel as well as individual public contributors. Having an established PPI group is an invaluable resource for a research team allowing researchers to respond quickly to changing research priorities. We would therefore encourage Clinical Trials Units who do not have access to public contributors to consider setting up their own panel. In countries and settings where PPI is less organised and structured, public contributors could be accessed through community-based organisations or using local media and social media to promote PPI opportunities.

The members of our Panel have varying levels of experience with PPI in clinical trials. Some PPI roles are better suited to contributors with previous experience in trials. However, providing feedback on early stage trial development relies heavily on experiential knowledge and we found that a lack of PPI trials experience was not a barrier to contributing to discussions.

In addition, this contribution is enhanced through the development of good working relationships both between the facilitators and PPI members, and between the members themselves which provides the optimal environment for rapid consultation work. Building strong relationships with public contributors is particularly important during the crisis as it is an anxious and stressful time for all and especially for those groups with underlying health conditions. The majority of members of our Panel have significant health issues and, against the background of the pandemic, the PPI process may be particularly anxiety-provoking for some contributors. PPI work conducted during this time offers opportunities to understand that anxiety, but also requires a mindful, respectful and emotionally sensitive approach in order to avoid any exacerbation of concerns.

The Evidence Base for Public Involvement in Clinical Trials (EPIC) study explores the different models of PPI that can be applied to a trial [14]. The report concludes that responsive and managerial roles for PPI contributors such as membership of Trial Management Groups (TMGs) are likely to bring greater benefit to the trial than PPI contributors in oversight positions such as Trial Steering Committees. At the time of working on RAPID-19, the CTU did not appoint PPI representatives to TMGs.

On reflection, we recognise that the study would have benefitted from having a PPI representative on the TMG to ensure continual PPI input throughout the development of the trial. Furthermore, the TMG PPI representative could advise the TMG on using the feedback from the Panel in adapting the trial protocol. As a result, the CTU is now looking to expand the models of PPI that we employ and are planning to appoint PPI representatives to sit on the TMG in future trials.

The feedback from the Panel could also be supplemented with a rapid review of existing studies with trials that explore relevant patient-acceptability themes applicable to the trial. Additionally, there is now a wealth of on-the-ground experience emerging from COVID-19 clinical trials. New trials would benefit from incumbent research teams sharing examples of good practice in PPI, and how they have overcome challenges to build knowledge and contribute to the evidence-base in this field.

Researchers have been sharing their experiences and best practice for online and remote co-production and patient involvement, as well as the pros and cons of the different virtual platforms available [15, 16]. Our PPI meeting was initially planned to be held by videoconference but some members of the Panel were not comfortable using digital platforms so we decided to conduct the meeting using teleconference with a freephone number. The meeting was lively and animated and prompted a lot of discussion. However, teleconferencing, unlike with videoconferencing, does not give you the benefit of non-verbal cues and it can be challenging to ensure that everyone in the group has an opportunity to speak. We followed up the meeting with an email to all attendees asking them to phone the facilitator or to send any further comments by email to ensure that we were able to benefit from the contribution of all attendees. Following the PPI panel meeting, the facilitator received one email and one phone call from Panel members wanting to provide further feedback on the trial as they had found it difficult to share their comments during the teleconference discussion.

Allowing PPI contributors to provide comment using various modes of communication recognises that individuals have different communication styles with some people preferring one-on-one conversation or giving written feedback rather than group discussion. Furthermore, encouraging PPI members to get in touch after a meeting gives contributors a period of reflection to develop thoughts that might be stimulated by discussion. Using WhatsApp, Microsoft Teams groups or other communication media would provide a forum for such discussion to continue after the meeting has ended. 

Our PPI Panel meeting was held early in the course of the pandemic when videoconferencing was not in common use. Since then, with the proliferation of digital platforms, people have become much more familiar with living and working virtually and increasingly PPI consultations are being conducted using videoconference. While there are inherent challenges with working virtually, it does offer opportunities. Even at the best of times, there are a number of difficulties with organising face-to-face meetings at short notice including the availability of some members and especially those with caring responsibilities, the cost of travel and the fact that our Panel is geographically dispersed across England. Virtual meetings can offer important solutions to these practical difficulties. In addition, many videoconferencing platforms allow meetings to be recorded which is helpful in producing detailed notes from the discussions. Before a meeting is recorded, researchers must obtain consent from all attendees and adhere to organisational General Data Protection Regulation policy [17]. Furthermore, new ways of working may help to broaden the representation of the patients and public involved in research and address the lack of diversity of PPI contributors which has long been recognised as a weakness of the PPI system [18].

We recognise there are costs involved for the Panel in terms of time and energy. Given the disproportionate impact of the pandemic on ethnic minorities, people from low socioeconomic groups and older adults, it is crucially important that these communities are supported to become actively involved in COVID-19 research. This will be critical for addressing the stark health inequalities exposed by the crisis. Particular attention should be given to the possibility that digital modalities might exclude certain communities from taking part in PPI. Researchers working with underrepresented groups must adopt a flexible approach and use a range of communication approaches to ensure inclusivity. 

PPI in research plays an important role in building public trust in scientific evidence [19]. A survey conducted by the Academy of Medical Sciences found that only a third of the public trusts evidence generated through medical research [19]. Crises create a climate of fear and panic, where misinformation and distrust can easily proliferate. Recent scandals during the pandemic, including the well-publicised retraction of the Lancet journal article on hydroxychloroquine [20] may further erode trust in science. Lack of trust may not only compromise recruitment into critical clinical trials, but, can also undermine the translation of that evidence into clinical practice. This is an opportune time to capitalise on the public’s heightened interest in research and science, and to build research literacy which leaves a lasting legacy to the wider society. Funders, Sponsors and Research Ethics Committees must uphold PPI standards for COVID-19 research including those for studies eligible for fast-track review.

## Limitations

This paper describes the lessons learnt for conducting rapid PPI consultation from the perspective of the research team. However, the PPI Panel have contributed to the manuscript and are named in the authorship of the paper. The findings presented in this paper could be strengthened by using qualitative research to actively explore with the PPI contributors, their experience of rapid PPI, the perceived impact of their involvement and to consider how they feel the process could be improved.

## Conclusion

The need for PPI is arguably more important now than ever. Conducting research in partnership with patients and members of the public will be critical for the successful delivery of COVID-19 clinical trials. The pandemic has accelerated change in clinical trials practices, driving trials and the review process to necessarily become more streamlined and efficient. The ability to conduct PPI in a time-efficient manner is not only essential for research during this crisis and for responding to future public health emergencies, but should also be applied beyond the pandemic.

We should leverage this newly-learned rapid engagement between researchers and public contributors, and the sharing of best practice developed during the COVID-19 crisis to create a new era of patient and public involvement for the benefit of future clinical trials research.

## Supplementary Information

**Additional file 1.** Daily participant diary. Daily patient diary for participants to use to monitor their symptoms.

## Data Availability

Not applicable.

## Abbreviations

ACEi: ACE Inhibitor
ARB: Angiotensin receptor blocker
CTU: Clinical Trials Unit
CVD: Cardiovascular disease
EPIC: Evidence Base for Public Involvement in Clinical Trials
GRIPP2: Guidance for Reporting Involvement of Patients and the Public
LSHTM: London School of Hygiene & Tropical Medicine
PPI: Patient and Public Involvement
REC: Research Ethics Committee
TMG: Trial Management Group
