# Supplementary material for: Patient and public involvement prior to trial initiation: lessons learnt for rapid partnership in the COVID-19 era
Source: Res Involv Engagem. 2021 Mar 8;7:13. doi: 10.1186/s40900-021-00250-9 (PMC7938674; doi:10.1186/s40900-021-00250-9)
Supplement: Supplementary file 1 — Additional file 1. Daily participant diary. Daily patient diary for participants to use to monitor their symptoms. [file 40900_2021_250_MOESM1_ESM.docx]

# DAILY PARTICIPANT DAIRY

| ***Prefilled Unique Participant ID*** | **Date:** |
| --- | --- |

**Medication question:**

Did you remember to take / not take your [insert ACEi / ARB name] today?

🞏 No | 🞏 Yes

**Symptoms questions:**

1. Do you have a fever? 🞏 No | 🞏 Yes

2. Please put a cross on the line below to indicate the severity of your cough:

0

No cough

100

Worst ever cough

3. Are you experiencing unusual shortness of breath? 🞏 No | 🞏 Yes

4. Are you experiencing unusual fatigue? 🞏 No | 🞏 Mild fatigue | 🞏 Severe fatigue – I struggle to get out of bed

5. Do you have a headache? 🞏 No | 🞏 Yes

6. Do you have a sore throat? 🞏 No | 🞏 Yes

7. Do you have a loss of smell / taste? 🞏 No | 🞏 Yes

8. Do you have an unusually hoarse voice? 🞏 No | 🞏 Yes

9. Do you have unusual abdominal pain? 🞏 No | 🞏 Yes

10. Are you experiencing diarrhoea? 🞏 No | 🞏 Yes

11. Do you have unusual strong muscle pains? 🞏 No | 🞏 Yes

12. Are there any other important symptoms you would like to share with us?

**Safety questions:**

13. Are you feeling an unusual chest pain or tightness in the chest? 🞏 No | 🞏 Yes, mildly | 🞏 Yes, frequently

14. Do you have unusual blurred or double vision? 🞏 No | 🞏 Yes, mildly | 🞏 Yes, frequently

15. Are you experiencing dizziness or vertigo? 🞏 No | 🞏 Yes, mildly | 🞏 Yes, frequently

If you answered “Yes, frequently” to question 13, 14 or 15, or are concerned for any reason, please contact the study team on:

**Breathing questions:**


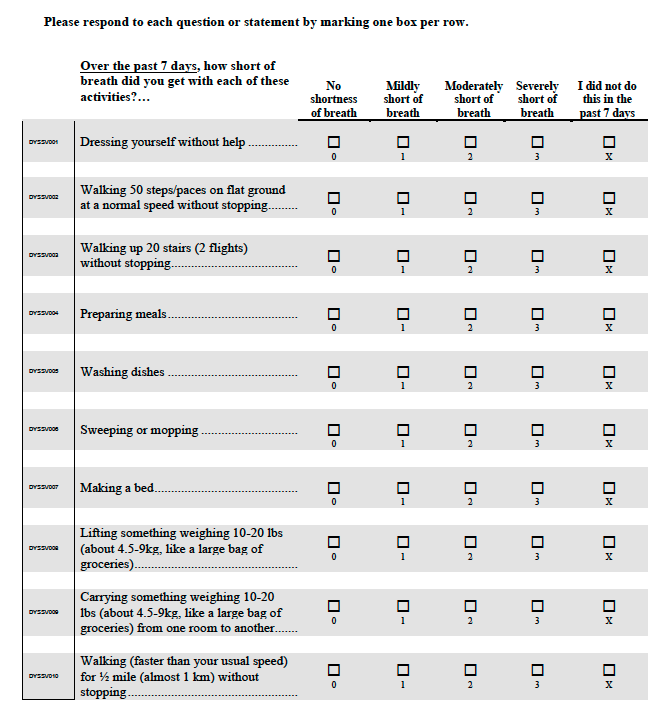
We would like you to tell us about your breathing, please could you answer the following questions:
